# A retrospective cohort analysis of people living with HIV/AIDS enrolled in HIV care at a reference center in Antananarivo, Madagascar

**DOI:** 10.3389/fpubh.2023.1329194

**Published:** 2024-01-15

**Authors:** Mihaja Raberahona, Rado Rakotomalala, Volatiana Andriananja, Johary Andriamamonjisoa, Etienne Rakotomijoro, Radonirina Lazasoa Andrianasolo, Rivonirina Andry Rakotoarivelo, Mamy Jean de Dieu Randria

**Affiliations:** ^1^Department of Infectious Diseases, University Hospital Joseph Raseta Befelatanana Antananarivo, Antananarivo, Madagascar; ^2^Faculty of Medicine, University of Antananarivo, Antananarivo, Madagascar; ^3^Department of Infectious Diseases, Faculty of Medicine, University Hospital Tambohobe Fianarantsoa, University of Fianarantsoa, Fianarantsoa, Madagascar

**Keywords:** retention in care, attrition, advanced HIV disease, risk factors, cohort, Madagascar

## Abstract

**Background:**

The impact of the “Treat all” policy on the individual and in terms of public health is closely related to early diagnosis and retention in care. Patient-level data are scarce in Madagascar. In this study, we aimed to describe the profile of a cohort of newly diagnosed people living with HIV/AIDS (PLHIV), identify their outcomes, and assess factors associated with attrition from care and advanced HIV disease (AHD) at presentation.

**Methods:**

We conducted a retrospective cohort study of PLHIV aged ≥15 years newly diagnosed at the University Hospital Joseph Raseta Befelatanana Antananarivo from 1 January 2010 to 31 December 2016.

**Results:**

A total of 490 PLHIV were included in the cohort analysis. In total, 67.1% were male. The median age (interquartile range) at enrollment in care was 29 years (24-38). Overall, 36.1% of PLHIV were diagnosed with AHD at baseline. The proportion of patients with WHO stage IV at baseline increased significantly from 3.3% in 2010 to 31% in 2016 (*p* = 0.001 for trend). The probability of retention in care after the diagnosis at 12 months, 24 months, and 36 months was 71.8%, 65.5%, and 61.3%, respectively. Age ≥ 40 years (aHR: 1.55; 95% CI: 1.05–2.29; *p* = 0.026), low level of education (aHR:1.62; 95% CI: 1.11–2.36; *p* = 0,013), unspecified level of education (aHR:2.18; 95% CI: 1.37–3.47; *p* = 0.001) and unemployment (aHR:1.52; 95% CI: 1.07–2.16; *p* = 0.019) were independently associated with attrition from care. Factors associated with AHD at baseline were age ≥ 40 (aOR: 2.77; 95% CI: 1.38–5.57, *p* = 0.004), unspecified level of education (aOR: 3.80; 95% CI: 1.58–9.16, *p* = 0.003) and presence of clinical symptoms at baseline (aOR: 23.81; 95% CI: 10.7–52.98; *p* < 0.001). Sex workers were independently less likely to have an AHD at presentation (aOR: 0.23; 95% CI: 0.05–0.96, *p* = 0.044).

**Conclusion:**

Sociodemographic determinants influenced retention in care more than clinical factors. The presence of clinical symptoms and sociodemographic determinants were the main factors associated with AHD at baseline.

## Introduction

In 2020, there were 42,000 (34,000–56,000) estimated people living with HIV/AIDS (PLHIV) and 6,000 (4,200–9,300) estimated newly infected people in Madagascar. However, only 6,700 (16%) actually knew their status, and 5,900 (88%) were on antiretroviral therapy (ART) (1). Thus, achieving the first 95 of the UNAIDS “95–95–95” targets by 2030 remains a challenge. Madagascar adopted a “Treat all” policy in late 2016, in line with the WHO guidelines. Early testing, early ART initiation, and long-term retention in care are essential to benefiting from the impact of the “Treat all” policy at the individual and public health levels (2). Cohort data on HIV/AIDS are scarce in Madagascar, especially on characteristics at enrollment in care and the long-term prognosis of PLHIV. A previous study showed a significant increase over time in hospital admissions of PLHIV for AIDS-related events. This suggests that PLHIV enrolled in care are still being diagnosed with AHD, as ~40% of PLHIV in that study were diagnosed during hospital admission (3). PLHIV presenting with AHD at enrollment in care are at high risk of hospitalization and death (4–7). In addition, AHD is still an independent risk factor for mortality, even in the virally suppressed cohort of PLHIV in Africa (8). Long-term retention in care of people enrolled in HIV care also remains a critical issue in low- and middle-income countries (9, 10). Despite the scaling-up in HIV testing and ART, only 52.1% of patients were retained on ART, and 41.8% were lost to follow-up (LTFU) in a study conducted in sub-Saharan Africa (11). PLHIV who are LTFU may contribute to up to 61% of new HIV infections (12). Evidence shows that virally suppressed PLHIV have an almost zero risk of transmitting HIV (13). Thus, retention in care of PLHIV already enrolled in care contributes significantly to reducing HIV transmission.

In this study, we aimed to describe the profile of a cohort of newly diagnosed PLHIV, identify their outcomes, and assess factors associated with attrition from care and AHD at presentation.

## Methods

### Study design and setting

We conducted a retrospective cohort analysis of routinely collected data on PLHIV enrolled in care at the Infectious Diseases Department of the University Hospital Joseph Raseta Befelatanana Antananarivo from 1 January 2010 to 31 December 2016. This department is a reference center and the largest HIV care facility in Madagascar and the Analamanga region. Point-of-care CD4 cell count is available on-site, and HIV viral load testing has been available since 2015 in collaboration with the Center d'Infectiologie Charles Mérieux in Antananarivo. Blood sampling for HIV viral load is carried out at the center. PLHIV are followed up by trained physicians, nurses, and social workers. ART is provided free of charge at the center according to national guidelines. The “Treat-all” policy has been applied since 2015, with a single-tablet efavirenz-based treatment used during the study period. ART is provided monthly, in most cases, by HIV nurses. Medical visits are scheduled every 6 months for PLHIV. Medical visits may be less or more frequent, depending on the clinical condition of the patient. CD4 cell count and HIV viral load testing are offered every 6 months according to the national guidelines, when available. Missing CD4 cell counts were caused by supply shortages or equipment malfunctions. When these situations occurred, testing was reserved for hospitalized or severely ill PLHIV.

We included in the study PLHIV of at least 15 years of age who were enrolled in care during the study period. The date of the data analysis was 10 October 2017. We did not apply any exclusion criteria. Missing data were not considered exclusion criteria and were included as a category in all variables.

### Outcome definition

PLHIV were considered LTFU if their last recorded visit was ≥180 days (14) as of 10 October 2017 and they were not clearly identified as dead or transferred to another HIV clinic in any medical records or visit logs. PLHIV were considered dead if there was a clear indication of their death, including the date of the event. Deaths of PLHIV may be recorded during hospital admission or reported later if the patient died elsewhere (in the community or other facilities).

PLHIV were considered to have been transferred to another HIV clinic if it was clearly stated in their medical records. Retention in care is defined as PLHIV not being LTFU, dead, or transferred to another HIV clinic at the time of data analysis. Attrition from care was defined as PLHIV who were LTFU or dead at the time of data analysis.

PLHIV were considered to have AHD at presentation if they had a CD4 cell count <200 per μL or were classified as stage 3 or 4 according to the WHO classification at baseline (15).

### Data collection

Data were collected from inpatient and outpatient medical records and visit logs of HIV care nurses and social workers in charge of psychosocial support. Standardized medical records provided by the national HIV program were available for outpatient follow-up. There is no electronic database, and all records and registries are in paper format. Data were collected from these different logs and then entered into an electronic case report form created with Epi Info 7 (CDC, Atlanta). To ensure patient confidentiality, each name was removed and replaced with a unique identifier. We did not collect home addresses, telephone numbers, or any other information that could lead to patient identification.

### Statistical analysis

Continuous variables were described with medians and interquartile ranges (IQR), whereas categorical variables were described as frequencies and percentages. Sociodemographic and clinical characteristics were described for the overall population and were stratified by year of enrollment in HIV care. The chi-square test or Fischer's exact test, as appropriate, was used to compare proportions, and the Wilcoxon rank-sum test was used to compare continuous variables. Trends over time periods were tested using the Cochran-Armitage trend test or the Jonckheere–Terpstra test, as appropriate. The probability of retention in care was estimated using the Kaplan-Meier method at 6, 12, 24, and 36 months. PLHIV who were transferred to other facilities for their follow-up were excluded from the survival analysis. Univariate and multivariate Cox proportional hazards regression was performed to determine factors independently associated with attrition from care. Covariates entered in the model were hypothesized a priori to influence attrition from care and were not limited to those with a significant statistical association with the outcome on univariate analysis. Age, sex, place of residence, marital status, sexual orientation, level of education, employment, point of entry into HIV care, disclosure to a sexual partner, HIV status of the sexual partner, and WHO stage at diagnosis were used in the model. The CD4 cell count was excluded from the model because of the large proportion of missing data and the unlikelihood of these being random. A missing data category was created for each covariate as needed. The proportional hazards assumption was checked with the Schoenfeld residuals test for each covariable and the final model. Covariables that did not fulfill the proportional hazards assumption were removed from the model (sex, sexual orientation, and point of entry into HIV care). Crude and adjusted hazard ratios (aHR) with 95% confidence intervals (95% CI) were determined from univariate and multivariate Cox proportional hazards regression models. We also assessed the factors associated with AHD at presentation in univariate and multivariate models using a binary regression logistic model. Variables with *P* < 0.1 were entered into the multivariate model, and variables with *P* < 0.05 were retained in the final model. A two-tailed *P* < 0.05 was considered significant. Statistical analysis was performed with Stata 14 (StataCorp LP).

### Ethical considerations

The collected data were anonymized to ensure patient confidentiality. All data extracted from medical records and visit logs were based solely on routinely collected data, and no additional data were collected for the purposes of the study. Therefore, the Malagasy National Ethics Committee waived the need for ethical approval and informed consent due to the retrospective design of the study and because we did not collect any further data other than those routinely collected (letter N°75 MSANP/CERBM).

## Results

### PLHIV characteristics at enrollment into HIV care

A total of 490 PLHIV were included in the analysis; 67.1% (*n* = 329) were men. The median age (IQR) at enrollment in care was 29 years (24-38). We did not find any differences in age at enrollment in care between male and female PLHIV, with a median age (IQR) of 30 years (24-37) vs. 28 years (25-38) (*p* = 0.552), respectively. Baseline characteristics at enrollment in HIV care are detailed in Table 1. A total of 30 (6.1%) PLHIV were enrolled in care in 2010, 20 (4.1%) in 2011, 34 (6.9%) in 2012, 80 (16.3%) in 2013, 94 (19.2%) in 2014, 106 (21.6%) in 2015, and 126 (25.7%) in 2016. There were significantly more women diagnosed with WHO stage IV than men (26.1% vs. 17.6%, *p* = 0.029). However, there were no differences in CD4 cell counts at enrollment in care between women and men. The total follow-up time was 1071.6 person-years. The median (IQR) follow-up time was 21 months (8-40). At the time of analysis, 112 (22.9%) were followed up for < 6 months, 41 (8.4%) for 6–11 months, 337 (68.8%) for ≥12 months, 231 (47.1%) for ≥24 months, and 143 (29.2%) for ≥36 months.

**Table 1:** Trends in baseline characteristics of PLHIV at enrollment in care.

	**Overall**	**2010**	**2011**	**2012**	**2013**	**2014**	**2015**	**2016**	***P*-value for trend**
	***n*** **(%)**	***n*** **(%)**	***n*** **(%)**	***n*** **(%)**	***n*** **(%)**	***n*** **(%)**	***n*** **(%)**	***n*** **(%)**	
Male patients	329 (67.1)	18 (60)	13 (65)	23 (67.6)	50 (62.5)	62 (66)	79 (74.5)	84 (66.7)	0.273
Age in years (median, IQR)	29 (24-38)	28 (24-32)	33 (27-39)	29 (25-31)	31 (24-35)	30 (24-36)	28 (24-36)	31 (25-43)	0.094
**Age group**									
•15–24	127 (22.2)	9 (30)	3 (15)	8 (23.5)	21 (26.3)	24 (25.5)	34 (32.1)	28 (22.2)	0.989
•25–39	266 (54.3)	19 (63.3)	15 (75)	22 (64.7)	45 (56.3)	53 (56.4)	55 (51.9)	57 (45.2)	**0.003**
•40–49	69 (14.1)	2 (6.7)	1 (5)	4 (11.8)	11 (13.8)	15 (16)	9 (8.5)	27 (21.4)	**0.032**
•≥50	28 (5.7)	0 (0)	1 (5)	0 (0)	3 (3.8)	2 (2.1)	8 (7.5)	14 (11.1)	**0.002**
**Place of residence**									
•City of Antananarivo	279 (56.9)	12 (40)	8 (40)	18 (52.9)	44 (55)	57 (60.6)	66 (62.3)	74 (58.7)	**0.019**
•Suburban area	97 (19.8)	6 (20)	3 (15)	8 (23.5)	12 (15)	17 (18.1)	26 (24.5)	25 (19.8)	0.555
•Outside Antananarivo	87 (17.8)	8 (26.7)	6 (30)	7 (20.6)	19 (23.8)	15 (16)	10 (9.4)	22 (17.5)	**0.020**
•Not documented	27 (5.5)	4 (13.3)	3 (15)	1 (2.9)	5 (6.3)	5 (5.3)	4 (3.8)	5 (4)	**0.026**
**Marital status**									
•Single	239 (48.8)	16 (53.3)	10 (50)	15 (44.1)	40 (50)	50 (53.2)	53 (50)	55 (43.7)	0.417
•Married	122 (24.9)	6 (20)	6 (30)	5 (14.7)	24 (30)	19 (20.2)	29 (27.4)	33 (26.2)	0.489
•Living together	56 (11.4)	1 (3.3)	1 (5)	10 (29.4)	8 (10)	10 (10.6)	11 (10.4)	15 (11.9)	0.834
•Divorced	20 (4.1)	1 (3.3)	1 (5)	0 (0)	1 (1.3)	7 (7.4)	4 (3.8)	6 (4.8)	0.375
•Widowed	10 (2)	1 (3.3)	0 (0)	0 (0)	2 (2.5)	1 (1.1)	2 (1.9)	4 (3.2)	0.534
•Not disclosed	43 (8.8)	5 (16.7)	2 (10)	4 (11.8)	5 (6.3)	7 (7.4)	7 (6.6)	13 (10.3)	0.429
**Sexual orientation**									
•Heterosexual	293 (59.8)	18 (60)	11 (55)	26 (76.5)	56 (70)	54 (57.4)	53 (50)	75 (59.5)	0.140
•Homosexual	68 (13.9)	4 (13.3)	5 (25)	2 (5.9)	6 (7.5)	16 (17)	17 (16)	18 (14.3)	0.557
•Bisexual	86 (17.6)	3 (10)	2 (10)	3 (8.8)	15 (18.8)	14 (14.9)	26 (24.5)	23 (18.3)	0.059
•Not disclosed	43 (8.8)	5 (16.7)	2 (10)	3 (8.8)	3 (3.8)	10 (10.6)	10 (9.4)	10 (7.9)	0.484
High level of education	183 (37.3)	15 (50)	5 (25)	19 (55.9)	24 (30)	33 (35.1)	42 (39.6)	45 (35.7)	0.385
**Employment**									
•Currently employed	330 (67.3)	22 (73.3)	13 (65)	21 (61.8)	51 (63.8)	65 (69.1)	74 (69.8)	84 (66.7)	0.921
•Student	62 (12.7)	4 (13.3)	1 (5)	2 (5.9)	8 (10)	10 (10.6)	19 (17.9)	18 (14.3)	0.115
•Unemployed	98 (20)	4 (13.3)	6 (30)	11 (32.4)	21 (26.3)	19 (20.2)	13 (12.3)	24 (19)	0.154
**Risk factors for HIV**									
•MSM	151 (30.8)	7 (23.3)	7 (35)	5 (14.7)	20 (25)	30 (31.9)	42 (39.6)	40 (31.7)	0.058
•Sex worker	31 (6.3)	0 (0)	1 (5)	0 (0)	6 (7.5)	7 (7.4)	7 (6.6)	10 (7.9)	0.086
•Injecting drug user	7 (1.4)	1 (3.3)	0 (0)	0 (0)	0 (0)	1 (1.1)	2 (1.9)	3 (2.4)	0.435
•Mobile worker	26 (5.3)	2 (6.7)	1 (5)	2 (5.9)	1 (1.3)	6 (6.4)	3 (2.8)	11 (8.7)	0.391
**Point of entry into care**									
•Clinical symptoms	239 (48.8)	12 (40)	7 (35)	17 (50)	50 (62.5)	39 (41.5)	48 (45.3)	66 (52.4)	0.521
•VCT	167 (34.1)	15 (50)	6 (45)	8 (23.5)	17 (21.3)	40 (42.6)	40 (37.7)	38 (30.2)	0.436
•PMTCT	23 (4.7)	3 (10)	1 (5)	3 (8.8)	6 (7.5)	2 (2.1)	4 (3.8)	4 (3.2)	**0.044**
•Infected sexual partner	43 (8.8)	0 (0)	0 (0)	4 (11.8)	4 (5)	12 (12.8)	11 (10.4)	12 (9.5)	0.062
•Other	18 (3.7)	0 (0)	3 (15)	2 (5.9)	3 (3.8)	1 (1.1)	3 (2.8)	6 (4.8)	0.892
**Disclosure to sexual partner**									
•Yes	99 (20.2)	5 (16.7)	3 (15)	11 (32.4)	14 (17.5)	17 (18.1)	23 (21.7)	26 (20.6)	0.799
•No	132 (26.9)	6 (20)	3 (15)	6 (17.6)	17 (21.3)	18 (19.1)	30 (28.3)	52 (41.3)	**< 0.001**
•Unknown	259 (52.9)	19 (63.3)	14 (70)	17 (50)	49 (61.3)	59 (62.8)	53 (50)	48 (38.1)	**< 0.001**
**HIV status of sexual partner**									
•Positive	62 (12.7)	1 (3.3)	0 (0)	6 (17.6)	6 (7.5)	14 (14.9)	17 (16)	18 (14.3)	**0.034**
•Negative	27 (5.5)	3 (10)	3 (15)	3 (8.8)	6 (7.5)	1 (1.1)	5 (4.7)	6 (4.8)	**0.045**
•Unknown	401 (81.8)	26 (86.7)	17 (85)	25 (73.5)	68 (85)	79 (84)	84 (79.5)	102 (81)	0.521
Diagnosis of HIV during hospitalization	95 (19.5)	0 (0)	4 (20)	3 (8.8)	20 (25)	19 (20.2)	17 (16)	32 (25.4)	**0.015**
**WHO stage at enrollment**									
•Stage I	282 (57.6)	22 (73.3)	13 (65)	21 (61.8)	38 (47.5)	57 (60.6)	61 (57.5)	70 (55.6)	0.222
•Stage II	31 (6.3)	1 (3.3)	1 (5)	4 (11.8)	6 (7.5)	7 (7.4)	5 (4.7)	7 (5.6)	0.678
•Stage III	77 (15.7)	6 (3.3)	5 (25)	6 (17.6)	14 (17.5)	12 (12.8)	24 (22.6)	10 (7.9)	0.067
•Stage IV	100 (20.4)	1 (3.3)	1 (5)	3 (8.8)	22 (27.5)	18 (19.1)	16 (15.1)	39 (31)	**< 0.001**
**CD4 counts at enrollment per** μ**L**									
•CD4 documented	337 (68.8)	20 (66.7)	9 (45)	23 (67.6)	68 (85)	67 (71.3)	91 (85.8)	59 (46.8)	0.068
• < 100	64 (19)	1 (5)	0 (0)	4 (17.4)	17 (25)	10 (14.9)	14 (15.4)	18 (30.5)	**0.047**
•100–199	54 (16)	1 (5)	1 (11.1)	4 (17.4)	11 (16.2)	13 (19.4)	12 (13.2)	12 (20.3)	0.276
• < 200	118 (35)	2 (10)	1 (11.1)	8 (34.8)	28 (41.2)	23 (34.3)	26 (28.6)	30 (50.8)	**0.013**
•200–349	92 (27.3)	10 (50)	3 (33.3)	5 (21.7)	19 (27.9)	19 (28.4)	25 (27.5)	11 (18.6)	**0.037**
•350–499	63 (18.7)	5 (25)	3 (33.3)	6 (26.1)	9 (13.2)	15 (22.4)	19 (20.9)	6 (10.2)	0.150
•≥500	64 (19)	3 (15)	2 (22.2)	4 (17.4)	12 (17.6)	10 (14.9)	21 (23.1)	12 (20.3)	0.428
AHD	204 (41.6)	8 (26.7)	6 (30)	11 (32.4)	42 (52.5)	37 (39.7)	45 (42.5)	55 (43.7)	0.163
Hepatitis B	30 (6.1)	5 (16.7)	0 (0)	4 (11.8)	7 (8.8)	5 (5.3)	4 (3.8)	5 (4)	**0.014**
Hepatitis C	3 (0.6)	0 (0)	1 (5)	0 (0)	1 (1.3)	0 (0)	0 (0)	1 (0.8)	0.468
Syphilis	20 (4.1)	3 (10)	0 (0)	1 (2.9)	3 (3.8)	2 (2.1)	2 (1.9)	9 (7.1)	0.816
**Outcome**									
•Retained	283 (57.8)	21 (70)	8 (40)	15 (44.1)	42 (52.5)	55 (58.5)	60 (56.6)	82 (65.1)	0.161
•Dead	54 (11)	1 (3.3)	2 (10)	5 (14.7)	7 (8.8)	9 (9.6)	13 (12.3)	17 (13.5)	0.167
•Loss to follow-up	129 (26.3)	6 (20)	9 (45)	12 (35.3)	26 (32.5)	23 (24.5)	30 (28.3)	23 (18.3)	0.053
•Transferred	24 (4.9)	2 (6.7)	1 (5)	2 (5.9)	5 (6.3)	7 (7.4)	3 (2.8)	4 (3.2)	0.208

MSM, Men who have sex with men; VCT, Voluntary testing and counseling; PMTCT, Prevention of Mother-to-Child Transmission. Bold values highlight statistical significant results.

### Trends in baseline characteristics

**Figure 1:**
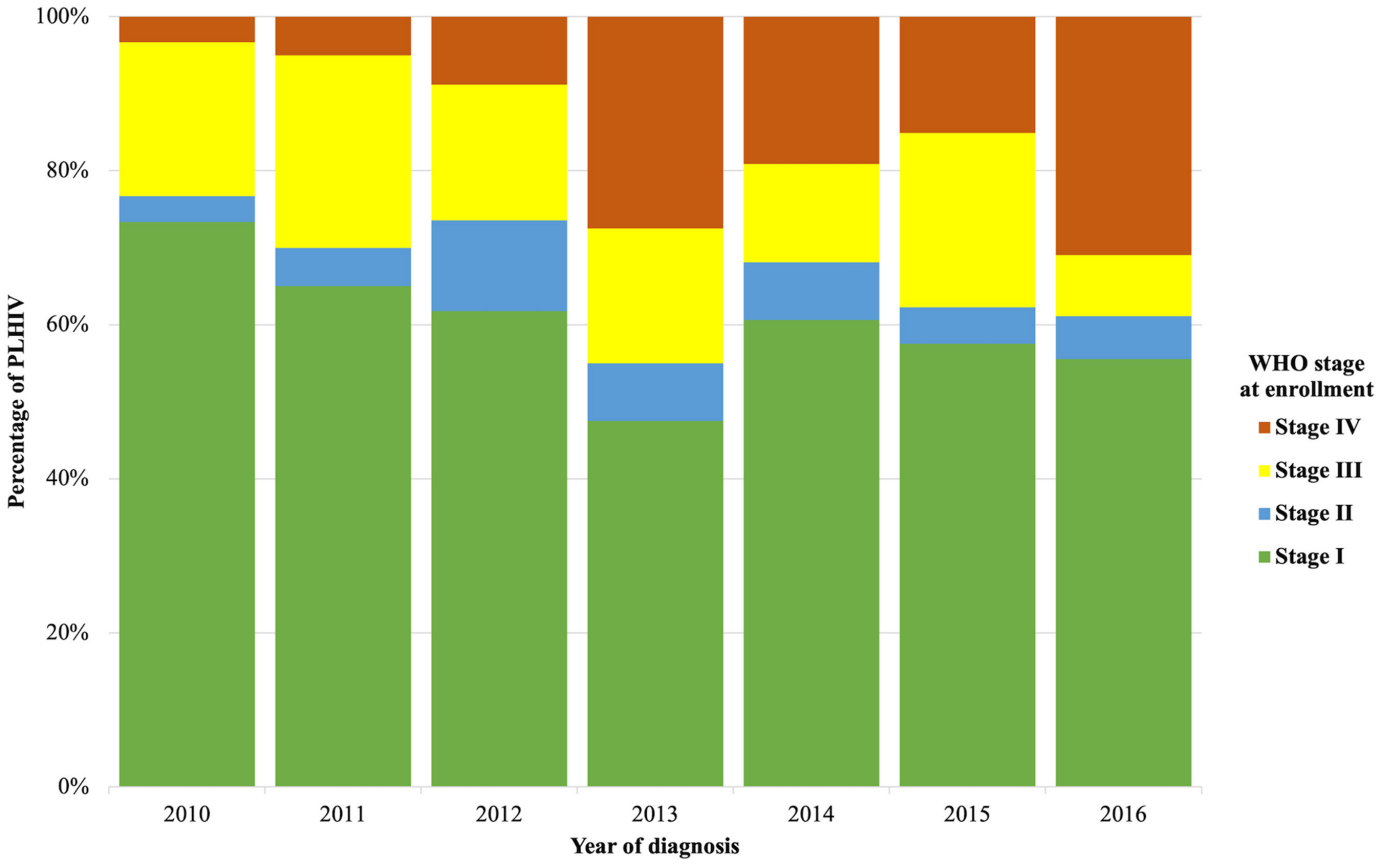
Trends in WHO stage at the time of diagnosis in PLHIV enrolement into care.

Trends in baseline characteristics at enrollment in HIV care from 2010 to 2016 are detailed in Table 1. From 2010 to 2016, the proportion of men newly diagnosed remained relatively stable (60% in 2010 vs. 66.7% in 2016, *p* = 0.273 for trend). From 2010 to 2016, there was a significant decrease in the proportion of PLHIV enrolled in care aged 25 to 39 years (63.3% in 2010 vs. 45.2% in 2016, *p* = 0.003 for trend), while the proportion of PLHIV aged 40 to 49 years (6.7% in 2010 vs. 21.4% in 2016, *p* = 0.032 for trend) and ≥50 years (0% in 2010 vs. 11.1% in 2016, *p* = 0.002 for trend) increased. We also noted an increase in PLHIV enrolled in care coming from the city of Antananarivo (40% in 2010 vs. 58.7% in 2016, *p* = 0.019 for trend). The proportion of PLHIV enrolled in care as part of the prevention of mother-to-child transmission (PMTCT) care decreased from 10% in 2010 to 3.2% in 2016 (*p* = 0.044 for trend). The proportion of PLHIV diagnosed during a hospital admission increased from 0% in 2010 to 25.4% in 2016 (*p* = 0.015 for trend). The proportion of PLHIV enrolled in care with WHO stage IV increased from 3.3% in 2010 to 31% in 2016 (*p* = 0.001 for trend) (Figure 1). We observed the same trend in the proportion of PLHIV enrolled in care with a CD4 cell count <200 per μl (10% in 2010 vs. 50.8% in 2016, *p* = 0.013 for trend) and <100 per μl (5% in 2010 vs. 30.5% in 2016, *p* = 0.047 for trend). The median CD4 cell count was 273 per μl (IQR: 136–437).

Overall, 36.1% of PLHIV were diagnosed with WHO stage III or IV, and this proportion remained stable over the study period (*p* = 0.143 for trend). There were significantly more PLHIV ≥40 years of age diagnosed with CD4 cell counts <200 per μL (35.6% vs. 10.9%, *p* < 0.001), CD4 cell counts <100 per μL (37.5% vs. 15.4%, *p* < 0.001), WHO stage III (33.8 vs. 17.2%, *p* = 0.001) and WHO stage IV (35% vs. 15.9%, *p* < 0.001) than those <40 years of age at diagnosis.

Overall, 41.6% (*n* = 204) of PLHIV were diagnosed with an AHD at enrollment in care, with no significant change over time.

There was no significant change in the proportion of PLHIV retained in care, dead, or lost to follow-up during the study period.

### Outcomes

Overall, 283 (57.8%) patients were retained in care, 54 (11%) were recorded as deceased, 129 (26.3%) were lost to follow-up, and 24 (4.9%) were transferred to other facilities. The total attrition from care was 183 (37.3%) at the time of analysis.

**Figure 2:**
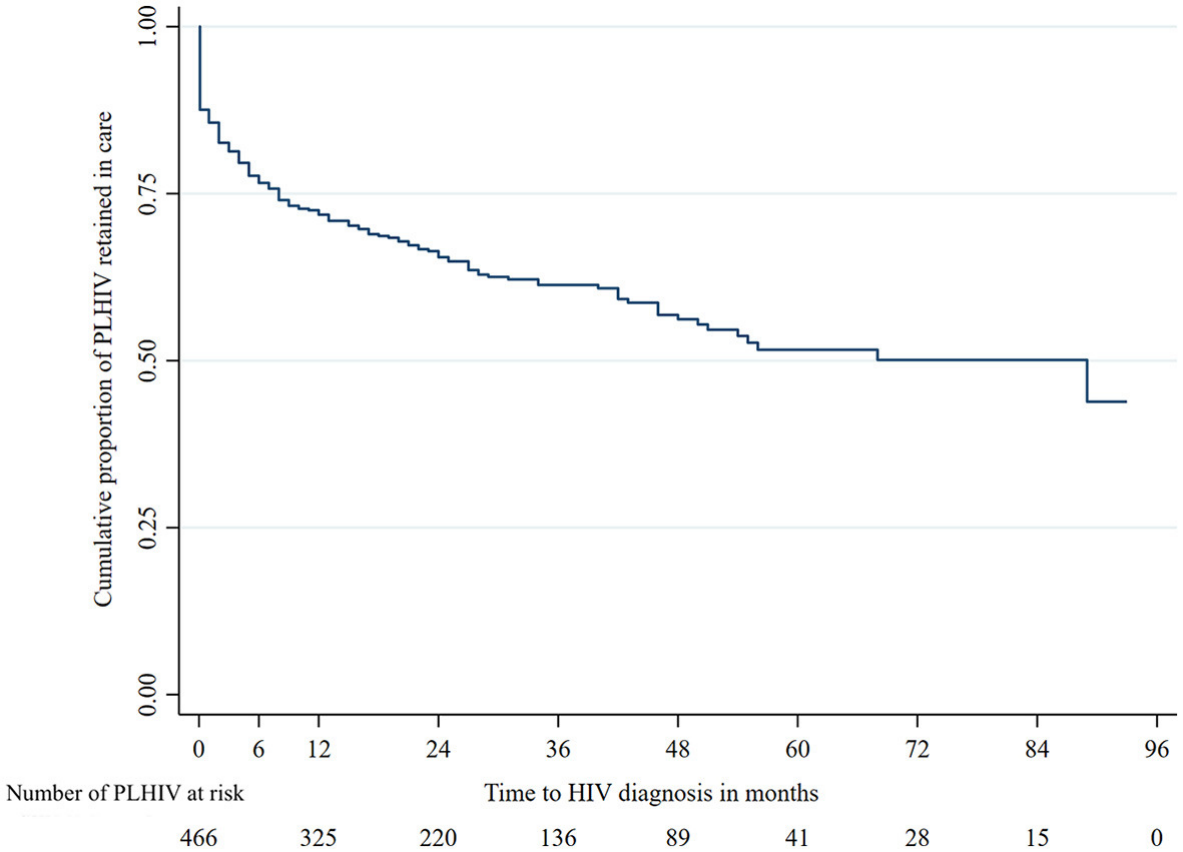
Kalpan–Meier curve estimating the probability of retention in care.

The probability of retention in care at 6, 12, 24, and 36 months was 76.6%, 71.8%, 65.5%, and 61.3%, respectively (Figure 2). The estimated median time of retention in care was 89 months (95% CI: 45.4–132.6). Attrition from care mostly occurred between 6 and 12 months following enrollment in care.

Among patients enrolled in care, 412 (84.1%) started ART with a median (IQR) delay of 23.5 (9-95) days. All patients retained in care started ART. Among patients who were LTFU or died, 110 (60.1%) had started ART.

### Factors associated with attrition from care

In the multivariate Cox proportional hazards regression model, we found that age ≥ 40 years (aHR: 1.55; 95% CI: 1.05–2.29, *p* = 0.026), low level of education (aHR: 1.62; 95% CI: 1.11–2.36, *p* = 0.013), unspecified level of education (aHR: 2.18; 95% CI: 1.37–3.47; *p* = 0.001), and unemployment (aHR: 1.52; 95% CI: 1.07–2.16; *p* = 0.019) were associated with attrition from care. Neither WHO stage nor HIV diagnosis during hospitalization at enrollment was associated with attrition from care when adjusting for other covariates of interest (Table 2).

**Table 2:** Factors associated with attrition from care.

**Variables**	**Unadjusted HR (95% CI)**	***P*-value**	**Adjusted HR (95% CI)**	***p*-value**
Age ≥ 40	1.52 (1.08–2.15)	0.017	**1.55 (1.05–2.29)**	**0.026**
**Place of residence**				
•Antananarivo	1		1	
•Outside Antananarivo	1.76 (1.25–2.48)	0.001	1.41 (0.98–2.03)	0.065
•Not documented	1.09 (0.57–2.08)	0.785	1.06 (0.54–2.08)	0.861
**Marital status**				
•Single	1		1	
•Married	0.95 (0.66–1.39)	0.808	0.82 (0.54–1.25)	0.362
•Other	1.06 (0.70–1.60)	0.798	1.05 (0.68–1.63)	0.813
•Not disclosed	2.46 (1.60–3.78)	< 0.001	1.41 (0.84–2.35)	0.194
**Education**				
•High level	1		1	
•Low level	1.55 (1.08–2.22)	0.017	**1.62 (1.11–2.36)**	**0.013**
•Unspecified level	3.03 (2.04–4.50)	< 0.001	**2.18 (1.37–3.47)**	**0.001**
**Employment**				
•Currently employed	1		1	
•Student	0.76 (0.45–1.28)	0.297	1.07 (0.61–1.88)	0.811
•Unemployed	1.68 (1.20–2.34)	0.002	**1.52 (1.07–2.16)**	**0.019**
**Disclosure to sexual partner**				
•Yes	1		1	
•No	1.65 (1.01–2.69)	0.046	1.11 (0.52–2.39)	0.788
•Unknown	1.96 (1.27–3.02)	0.002	1.01 (0.47–2.15)	0.985
**HIV status of sexual partner**				
•Positive	1		1	
•Negative	1.01 (0.40–2.53)	0.985	0.93 (0.36–2.36)	0.873
•Unknown/Untested	2.10 (1.19–3.70)	0.010	1.81 (0.74–4.41)	0.192
**WHO stage at enrollment**				
•Stage I	1		1	
•Stage II	0.77 (0.37–1.58)	0.470	0.67 (0.32–1.41)	0.290
•Stage III	0.99 (0.65–1.51)	0.973	0.73 (0.46–1.15)	0.174
•Stage IV	2.01 (1.43–2.84)	< 0.001	1.09 (0.64–1.85)	0.744
HIV diagnosed during hospitalization	2.02 (1.46–2.81)	< 0.001	1.30 (0.79–2.16)	0.305

Bold values highlight statistical significant results.

### Factors associated with AHD at presentation

In logistic multivariate analysis, age ≥ 40 (aOR: 2.77; 95% CI: 1.38–5.57, *p* = 0.004), unspecified level of education (aOR: 3.80; 95% CI: 1.58–9.16, *p* = 0.003) and presence of clinical symptoms at baseline (aOR: 23.81; 95% CI: 10.7–52.98; *p* < 0.001) were associated with advanced HIV diagnosis (Table 3). Sex workers were found to be independently less likely to have an AHD at presentation (aOR: 0.23; 95% CI: 0.05–0.96, *p* = 0.044).

**Table 3:** Factors associated with AHD at presentation.

**Variables**	**Unadjusted OR (95% CI)**	***p*-value**	**Adjusted OR (95% CI)**	***p*-value**
Age ≥ 40^¶^	4.17 (2.59–6.73)	< 0.001	**2.77 (1.38–5.57)**	**0.004**
Sex: male	0.89 (0.61–1.31)	0.562		
**Place of residence** ^¶^				
•Antananarivo	1		1	
•Outside Antananarivo	1.94 (1.21–3.11)	0.006	0.98 (0.50–1.91)	0.948
•Not documented	0.66 (0.54–0.82)	0.034	0.43 (0.12–1.62)	0.214
**Marital status** ^¶^				
•Single	1		1	
•Married	2.72 (1.73–4.26)	< 0.001	1.52 (0.72–3.19)	0.268
•Other	1.26 (0.75–2.11)	0.388	0.94 (0.42–2.09)	0.882
•Not documented	6.49 (3.10–13.57)	< 0.001	0.97 (0.30–3.11)	0.963
**Education** ^¶^				
•High level	1		1	
•Low level	0.77 (0.51–1.16)	0.201	1.20 (0.67–2.16)	0.544
•Unspecified level	4.55 (2.57–8.05)	< 0.001	**3.80 (1.58–9.16)**	**0.003**
**Employment** ^¶^				
•Student	1		1	
•Currently employed	1.87 (1.04–3.37)	0.037	1.10 (0.49–2.47)	0.809
•Unemployed	1.91 (0.97–3.77)	0.061	1.05 (0.39–2.82)	0.917
**Sexual orientation** ^¶^				
•Homosexual	1		1	
•Heterosexual	3.39 (1.80–6.37)	< 0.001	1.60 (0.10–25.66)	0.742
•Bisexual	1.67 (0.79–3.53)	0.177	1.66 (0.64–4.27)	0.295
•Not disclosed	6.51 (2.77–15.28)	< 0.001	2.34 (0.12–45.27)	0.574
**Risk factors for HIV**				
•MSM^¶^	0.37 (0.24–0.56)	< 0.001	0.67 (0.04–11.08)	0.777
•Sex worker^¶^	0.14 (0.04–0.46)	0.001	**0.23 (0.05–0.96)**	**0.044**
•Injecting drug user	1.05 (0.23–4.75)	0.947	-	-
•Mobile worker	1.43 (0.65–3.15)	0.375	-	-
**Point of entry into care**				
•VCT^¶^	0.08 (0.04–0.13)	< 0.001	1.22 (0.48–3.06)	0.678
•Clinical symptoms^¶^	25.25 (15.33–41.61)	< 0.001	**23.81 (10.70–52.98)**	**< 0.001**
•PMTCT	0.12 (0.03–0.54)	0.005	-	-
•Infected sexual partner	0.20 (0.08–0.49)	< 0.001	-	-
•Other	0.18 (0.04–0.79)	0.023	-	-

MSM, Men who have sex with men; VCT, Voluntary testing and counseling; PMTCT, Prevention of Mother-to-Child Transmission. ^¶^Variables entered in the multivariate model. Bold values highlight statistical significant results.

## Discussion

In this retrospective cohort study, we found that 67.1% of the PLHIV diagnosed from 2010 to 2016 were male, with no significant changes over the study period. The proportion of PLHIV aged 25 to 39 years slightly decreased over the study period, while the proportion of PLHIV aged 40 to 49 years and those aged ≥ 50 years increased significantly. Newly diagnosed PLHIV coming from outside the city of Antananarivo decreased significantly over the study period. There were no significant changes in the sociodemographic parameters of newly diagnosed PLHIV regarding marital status, sexual orientation, educational level, or employment status. There was a significant decline in PMTCT as a point of entry into care. From a clinical perspective, there were significant increases in the proportion of PLHIV diagnosed with HIV during a hospital admission, the proportion of PLHIV diagnosed at WHO stage IV, and the proportion of PLHIV diagnosed with CD4 cell counts < 200 per μL and < 100 per μL at enrollment in care. At 6 months and 12 months following enrollment in care, the probability of retention in care was 76.6% and 71.8%, respectively, with the majority of the attrition from care occurring during this period. Overall, 57.8% of PLHIV enrolled in care were retained. We found that age, level of education, and unemployment were associated with attrition from care.

This study has several limitations due to its retrospective design, mainly because of missing data, which may reduce the power to detect factors associated with attrition from care. Its monocentric design may also limit its generalizability. However, in Madagascar, detailed data from other centers are generally scarce due to the absence of a well-organized multicenter prospective cohort data registry at the national level. We also acknowledge that the sociodemographic information provided by PLHIV at enrollment for care may not be as accurate as needed and could not be further verified because it was based on their self-report only. This is particularly true for information related to sexual orientation, marital status, or place of residence, due to privacy concerns or perceived stigma. CD4 cell counts were not available for all patients due to frequent disruptions in reagent supply during the study period. More recent data would have provided a more comprehensive understanding of the current situation. Nevertheless, analyzing cohort data in HIV care centers in Madagascar remains challenging due to the lack of centralized and electronic medical records. PLHIV who are being transferred to a different facility must have a transfer form provided by their current HIV care center addressed to the new HIV care center. Therefore, a self-transferred PLHIV (a PLHIV who transfers to a new HIV care center without informing their initial HIV care center) will be misclassified as LTFU at their initial HIV care center. In the absence of a centralized national database, it is virtually impossible to accurately check this information. The same is true for undocumented deaths. These issues are known in PLHIV cohorts. A systematic review and meta-analysis estimated that 18.6% (95% CI: 15.8%−22%) of PLHIV classified as LTFU were actually self-transferred and 38.8% (95% CI: 30.8%−46.8%) were actually dead (16). Another study found that the estimated proportion of PLHIV retained on ART was 66.6%, compared to 52.1% in the crude analysis (14.1% absolute difference) when self-transfers and undocumented deaths were included (11). Therefore, this study provides a more conservative estimate of the retention and attrition from care by using a worst-case scenario model.

In contrast to cohorts from other African regions and African countries, where two-thirds of PLHIV are women (17–19), this cohort is predominantly male. Data from the National HIV/AIDS Program in 2022 showed that of 10,836 PLHIV followed up in Madagascar, 42% were men. In 2021, ~70% of new HIV infections in Madagascar consisted of women. However, national data also showed that PLHIV in the Analamanga region, the region where the center for this study is located, were predominantly men. This preponderance remains even when the data from the setting of this cohort study are removed, supporting that it is not influenced by data from a large center. A possible explanation is the difference between the predominant modes of HIV transmission in different regions of Madagascar.

AHD is still a major concern in Africa, despite widespread access to ART (20). In African countries, the proportion of PLHIV starting ART with CD4 counts <200 per μL ranged from 34% to 75% for female patients from 2005 to 2012 and from 41% to 80% for male patients in the same period in one study (20). However, an improvement in the median CD4 cell count at ART initiation was noted in low-income countries that included mostly African countries from 2005 to 2012 (20). Later, a prospective cohort study conducted in 2017–2018 in Senegal showed that the proportion of PLHIV starting ART with AHD was still high (21). Nevertheless, large cohort data from southern Africa showed a decrease in the proportion of PLHIV starting ART with AHD between 2005 and 2018 (22). In fact, 83.3% of PLHIV presented with AHD in 2005, whereas this proportion fell to 23.5% in 2018 (22). This proportion of PLHIV diagnosed with AHD seems to plateau at approximately one-third of PLHIV, even in the context of high ART coverage (23). However, our study highlighted that HIV was still diagnosed at AHD in this cohort of PLHIV with no significant improvement between 2010 and 2016. Several lines of evidence from this cohort support these findings about worsening situations over time.

Recent studies in other high-burden African countries have also shown a shift in HIV incidence toward those who are older, rather than the traditional young age group, which is the primary target of HIV prevention strategies (24, 25). Although the HIV epidemic profile is different in Madagascar, there is likely to be a need to expand the target of prevention and even more testing strategies to include people of an older age, as they are particularly at risk of being diagnosed late.

In this study, we observed that the proportion of PLHIV coming from outside the region of Antananarivo decreased over time. This is likely due to the increase in the number of centers able to provide care and ensure a long-term follow-up of PLHIV in recent years. However, despite this progress, our center still ensures the follow-up and dispensing of ART to PLHIV coming from other regions. In fact, some PLHIV prefer to be treated and followed up in centers located in large urban areas because of concerns about confidentiality that arise if they are followed up in centers located in smaller towns where people know each other well. This is often reported by PLHIV living in regions outside of Antananarivo and followed up at our center. Since 2018, hundreds of physicians have been trained and deployed at the district level. However, the perception of stigma by PLHIV can be greater in smaller communities, particularly in small or isolated towns, and can be a barrier to a community-based approach to testing and care strategies for PLHIV.

This study showed that most attrition from care occurs in the first year following enrollment in care. The first year after enrollment is critical for retention, with a non-negligible proportion of patients dropping out shortly after enrollment into care.

Risk factors for LTFU can be divided into sociodemographic, clinical, behavior-related, treatment-related, and system-level determinants (26). In this study, we only explored sociodemographic and clinical determinants. We have shown that retention in care is mainly influenced by sociodemographic and economic factors, including age, level of education, and employment, rather than clinical factors at baseline, such as the WHO clinical stage. A systematic review showed that demographic factors such as age, sex, marital status, and employment status were important factors for loss to follow-up in addition to clinical factors such as low CD4 cell count, tuberculosis, and opportunistic infections (27). However, one study did not find any association between attrition from care and CD4 cell count or tuberculosis at enrollment in care (26). In developed countries, demographic factors and substance use are the most frequently reported factors for poor retention in care, while clinical factors such as HIV progression and comorbidities are the most frequently cited in developing countries (28). In our cohort, even as the proportion of patients enrolled in care with AHD, with a high probability of various opportunistic infections and tuberculosis, increased, these clinical factors were less predictive of retention in care compared to other sociodemographic and economic factors when adjusted for other factors in the multivariate model. A study conducted in South Africa showed that nearly 40% of PLHIV will disengage from care by 12 months after HIV diagnosis (29). This study also showed that attrition from care was substantially improved by 30% when a universal test and treatment policy were applied (29). Nevertheless, another study showed that the implementation of this strategy has successfully improved the proportion of PLHIV entering into care at an early stage of HIV, but conversely increased attrition from care (30). As the period considered in our cohort study ended a few months after the implementation of the “Treat all” strategy in Madagascar, the impact of this strategy on attrition from care remains unclear. Furthermore, long-term retention in care remains a challenge, with a cumulative incidence of attrition from care of up to 75% 10 years after ART initiation among adult PLHIV in a study in Africa (31).

## Conclusion

Overall, one-third of PLHIV were diagnosed with AHD, but the proportion of PLHIV diagnosed with WHO stage IV or with CD4 counts <200 per μL increased in the last years of the study period. Approximately half of the PLHIV presented clinical symptoms at baseline, and only one-third were diagnosed through voluntary testing and counseling. The probability of retention in care at 6, 12, 24, and 36 months was 76.6%, 71.8%, 65.5%, and 61.3%, respectively, with the majority of attrition from care occurring in the first 12 months following entry into care. Factors associated with attrition from care were age, level of education, and professional situation. Factors associated with AHD were age, level of education, and the presence of clinical symptoms at baseline.

Current evidence suggests that PLHIV in our cohort are still increasingly diagnosed with AHD. Retention in care was mostly influenced by sociodemographic determinants rather than clinical determinants.

## Data availability statement

The original contributions presented in the study are included in the article/Supplementary material, further inquiries can be directed to the corresponding author.

## Ethics statement

The study was conducted in accordance with the local legislation and institutional requirements. The Malagasy National Ethics Committee of the Ministry of Public Health (CERBM) waived the need for ethics approval and informed consent due to the retrospective design of the study and because we did not collect any further data other than those routinely collected (letter N°75/MSANP/CERBM).

## Author contributions

MR: Conceptualization, Data curation, Formal analysis, Investigation, Methodology, Supervision, Validation, Writing – original draft, Writing – review & editing. RR: Conceptualization, Investigation, Methodology, Writing – original draft. VA: Conceptualization, Investigation, Writing – review & editing. JA: Conceptualization, Investigation, Writing – review & editing. ER: Conceptualization, Investigation, Writing – review & editing. RA: Conceptualization, Investigation, Supervision, Writing – review & editing. RAR: Conceptualization, Investigation, Methodology, Supervision, Writing – review & editing. MJdDR: Conceptualization, Investigation, Methodology, Supervision, Writing – review & editing.
